# Cytogenetics Meets Genomics: Cytotaxonomy and Genomic Relationships among Color Variants of the Asian Arowana *Scleropages formosus*

**DOI:** 10.3390/ijms24109005

**Published:** 2023-05-19

**Authors:** Gustavo A. Toma, Natália dos Santos, Rodrigo dos Santos, Petr Rab, Rafael Kretschmer, Tariq Ezaz, Luiz A. C. Bertollo, Thomas Liehr, Fábio Porto-Foresti, Terumi Hatanaka, Alongklod Tanomtong, Ricardo Utsunomia, Marcelo B. Cioffi

**Affiliations:** 1 Departamento de Genética e Evolução, Universidade Federal de São Carlos, São Carlos 13565-905, SP, Brazil; gustavo_toma@hotmail.com (G.A.T.); bertollo@ufscar.br (L.A.C.B.); hterumi@yahoo.com.br (T.H.); mbcioffi@ufscar.br (M.B.C.); 2 Faculdade de Ciências, UNESP, Bauru 17033-36, SP, Brazil; n.santos97@unesp.br (N.d.S.); rodrigo.zeni@unesp.br (R.d.S.); fp.foresti@unesp.br (F.P.-F.); ricardo.utsunomia@unesp.br (R.U.); 3 Laboratory of Fish Genetics, Institute of Animal Physiology and Genetics, Czech Academy of Sciences, Rumburská 89, 27721 Liběchov, Czech Republic; rab@iapg.cas.cz; 4 Departamento de Ecologia, Zoologia e Genética, Universidade Federal de Pelotas, Pelotas 96010-900, RS, Brazil; rafa.kretschmer@hotmail.com; 5 Institute for Aplied Ecology, University of Canberra, Canberra 2617, Australia; tariq.ezaz@canberra.edu.au; 6 Institute of Human Genetics, University Hospital Jena, 07747 Jena, Germany; 7 Department of Biology, Faculty of Science, Khon Kaen University, Muang, Khon Kaen 40002, Thailand; tanomtong@hotmail.com

**Keywords:** Osteoglossiformes, cytogenomics, chromosome, molecular cytogenetics, SatDNA

## Abstract

*Scleropages formosus* (Osteoglossiformes, Teleostei) represents one of the most valued ornamental fishes, yet it is critically endangered due to overexploitation and habitat destruction. This species encompasses three major color groups that naturally occur in allopatric populations, but the evolutionary and taxonomic relationships of *S. formosus* color varieties remain uncertain. Here, we utilized a range of molecular cytogenetic techniques to characterize the karyotypes of five *S. formosus* color phenotypes, which correspond to naturally occurring variants: the red ones (Super Red); the golden ones (Golden Crossback and Highback Golden); the green ones (Asian Green and Yellow Tail Silver). Additionally, we describe the satellitome of *S. formosus* (Highback Golden) by applying a high-throughput sequencing technology. All color phenotypes possessed the same karyotype structure 2n = 50 (8m/sm + 42st/a) and distribution of SatDNAs, but different chromosomal locations of rDNAs, which were involved in a chromosome size polymorphism. Our results show indications of population genetic structure and microstructure differences in karyotypes of the color phenotypes. However, the findings do not clearly back up the hypothesis that there are discrete lineages or evolutionary units among the color phenotypes of *S. formosus*, but another case of interspecific chromosome stasis cannot be excluded.

## 1. Introduction

Osteoglossidae (Osteoglossiformes, Teleostei) encompasses two reciprocally monophyletic clades, the Arapaiminae and the Osteoglossinae, the latter being commonly known as “arowanas” [1,2], which includes two genera: *Osteoglossum*, in South America, and *Scleropages*, in Australasia [1,2,3]. The *Scleropages* genus comprises four extant species: *S. leichardti* Günther, 1864 and *S. jardinii* Saville-Kent, 1892, found in the Sahul region, and the two remaining ones found in South Asia [1,3], namely, *S. inscriptus* Roberts, 2012, in Myanmar, and *S. formosus* Müller and Schlegel, 1840, in the Malay peninsula, Sumatra, and western parts of Kalimantan [2,3,4,5].

*S. formosus*, known as Asian arowana or dragon fish, stands out for its distinctive color variations, which range from bright red to lustrous silvery-green, being one of the most highly prized and expensive species of ornamental fish, with commercial values topping USD 20,000 per individual [6,7]. As a result, the natural populations of *S. formosus* are highly exploited, and they are listed among endangered species [8].

*S. formosus* typically comprises three naturally distinct color morphs: (1) the red variety, which includes phenotypes known as Chili Red, Blood Red, and Super Red, and (2) the golden variety, which includes the phenotype known as Indonesian Golden or Red Tail Golden, as well as the phenotype known as Malaysian Golden, which is further subdivided into Blue-based Golden, Highback Golden, and Golden Crossback, and (3) the green variety, which includes phenotypes known as Yellow Tail Silver and Asian Green [6,7,9,10]. The red variety is distributed throughout western Borneo (upstream of Kapuas River, Kalimantan—Indonesia), the golden variety is found in Indonesia (Sumatra Island) and Malaysia (Perak), and the green variety has a broader distribution across Vietnam, Myanmar, Cambodia, Thailand, Malaysia, and Indonesia [7,10,11,12]. Therefore, the red and golden varieties are allopatric, whereas the green variety, with a broad distribution, is sympatric with the red and golden ones [7,10,11,12]. The evolution of these three major varieties remains unclear, although some authors have suggested that they had an independent origin in different Southeast Asia regions during the Pleistocene glaciations [13,14].

Data from mitochondrial DNA (cytochrome B) and morphological analyses suggested that some *S. formosus* color variants could represent distinct species [14], a pattern not supported by the studies of [15]. In fact, investigations still remains without a complete concordance along the time. Kottelat [16] supported the species status for *S. legendrei* (Super red phenotype). In turn, Mohd-Shamsudin et al. [9] compared specimens of the three main varieties (red, golden, and green) of *S. formosus* using two mitochondrial DNA markers—cytochrome B and cytochrome oxidase subunit I—suggesting that they are not consistent with a species status, probably representing a single species. Two additional studies using mitochondrial DNA [17] and the 18S ribosomal gene [18] showed similar results. The most recent study with two mitochondrial and one nuclear gene in a dense geographical sample indicated that the phylogenetic lineages of *S. formosus* are better explained by geographic paleodrainages than by their color varieties [12]. As a result, the evolutionary and taxonomic relationships of *S. formosus* color varieties remain uncertain.

In this regard, modern cytogenetic tools, such as mapping of repetitive DNAs, comparative genome hybridization (CGH), and whole chromosome painting (WCP), have been shown as prominent approaches for investigating ancient fish lineages [19,20,21,22,23]. These approaches allow us to draw comparisons between related species or population genomes, helping to better highlight the evolutionary paths of distinct karyotypes, both at intra- and interspecific levels [24,25]. Moreover, the integration of cytogenetics with other new procedures, such as high-throughput sequencing (Next Generation Sequencing—NGS) and in-depth pipelines, provides researchers with a more refined look into the genomic organization of chromosomes, especially of its repetitive fraction. As a result, it is possible to achieve the assembly of many high-quality repetitive DNA libraries, even in non-model organisms lacking reference genomes [26,27,28]. The satellitome, which represents a catalog of satellite DNAs (SatDNAs), is one of these novel DNA libraries [28]. Head-to-tail units of a single DNA sequence, sometimes referred to as a monomer, make up these repetitive DNAs. Recent studies have shown the substantial effects of the SatDNA framework on the evolutionary biology of various animal species [29,30,31]. It is considered that some species–specific sequences and very conserved satellite families are related to population dynamics, speciation processes, and B- and sex chromosome evolution [26,32,33,34].

In this study, part of a series on cytogenetics and genomics of Osteoglossiformes fishes, we describe the karyotype of five *S. formosus* phenotypes, encompassing the three naturally occurring varieties: red (Super Red), golden (Golden Crossback and Highback Golden), and green (Asian Green and Yellow Tail Silver). In addition, we used a selection of cytogenetic methods, including Giemsa staining, C-banding, repetitive DNA mapping, comparative genome hybridization (CGH), and whole chromosome painting (WCP) to highlight possible karyotype differences in *S. formosus*. Finally, we addressed the *S. formosus* (Highback Golden variety) satellitome by utilizing a high-throughput sequencing platform and the TAREAN pipeline.

## 2. Results

### 2.1. SatDNA Content of S. formosus Genome

**Figure 1:**
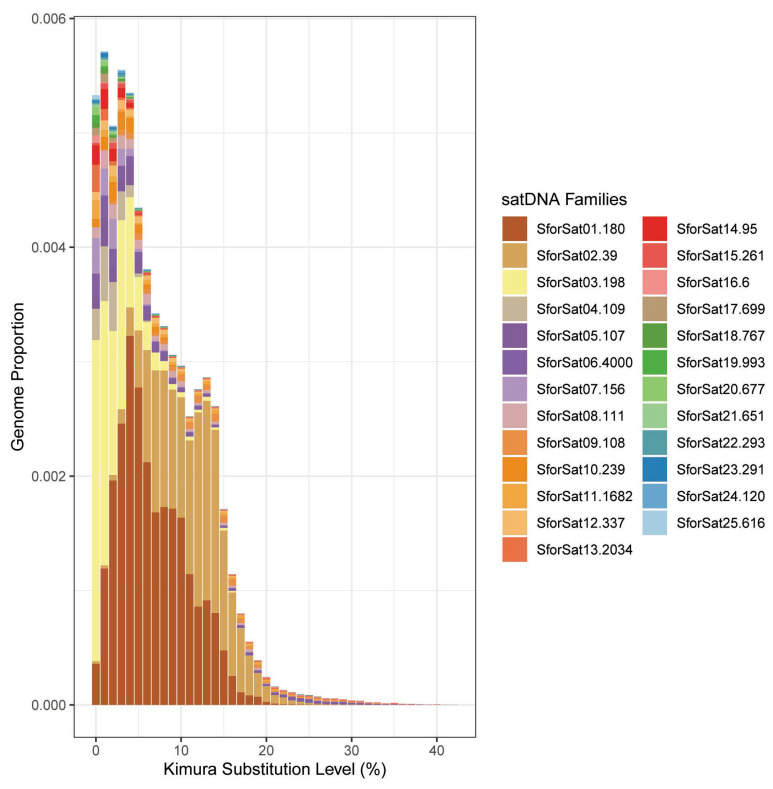
Repeat landscape showing the abundance (Y axis) and Kimura-2-divergence (X axis) profiles for all SatDNAs identified in *S. formosus*.

Low-coverage shotgun genome sequencing data from a single individual was used in repeat clustering with TAREAN, which, after 5 iterations, resulted in 25 SatDNA families for *S. formosus* (Figure 1). The A + T was greater than 50% in 21 SatDNAs families. The repeat unit lengths (RUL) ranged from 6 to 4000 bp, with a median of 261 bp (Table 1). The length distribution of the SatDNA families showed that long (>100 bp) were prevalent, with 22 SatDNA families included in this category. The search for homology between the sequences of the SatDNA families revealed the occurrence of one superfamily, (SfoSat21-651 and SfoSat23-291), with 57% of local similarity. The BLAST search against GenBank/NCBI databases revealed no significant similarity for any SfoSat DNAs.

### 2.2. Karyotypes and C-Banding

**Figure 2:**
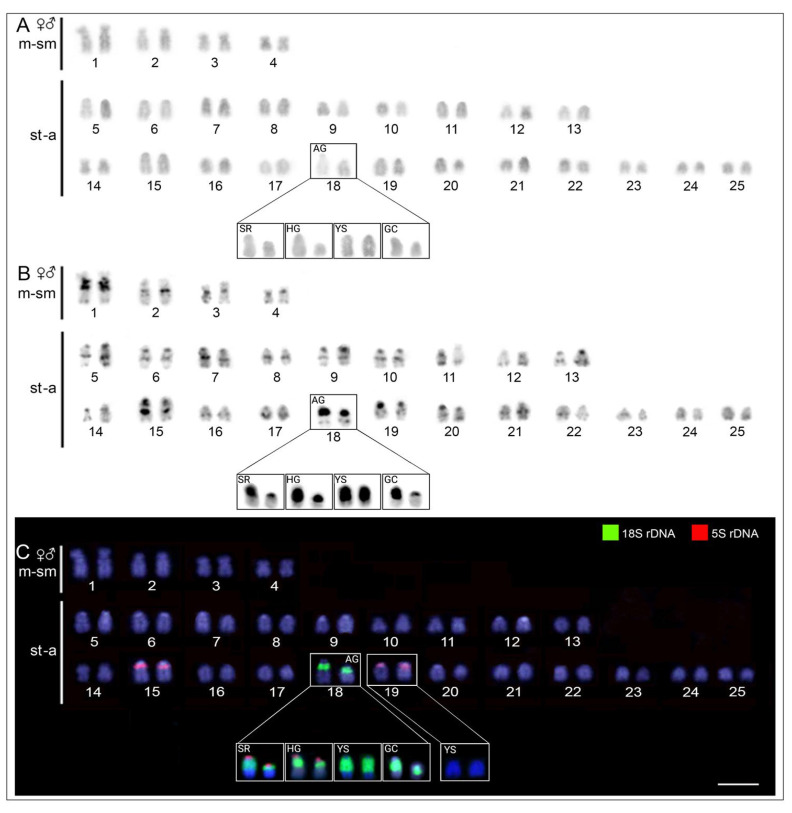
Karyotypes of *S. formosus* phenotypes (males and females) here represented by metaphases of the AG phenotype, arranged from Giemsa-staining (**A**), C-banded chromosomes (**B**), and mapping of 18S (green) and 5S (red) rDNA probes (**C**). In boxes (**A**–**C**), the polymorphic 18steoglossiformes pair was highlighted along with the YS 19steoglossiformes pair, which does not bear 5S rDNA sites. Bar = 5 μm.

All five *S. formosus* color phenotypes had the same 2n = 50 and karyotypes composed of 8m/sm + 42st/a chromosomes in both females and males (Figure 2; Supplementary Figure S1), without indications of heteromorphic sex chromosomes. However, we found a morphological polymorphism in the 18th chromosome pair, ranging between a large homomorphic acrocentric pair in YS and a heteromorphic pair with relatively large and small acrocentric chromosomes in AG, SR, HG, and GC phenotypes (highlighted inside the boxes of Figure 2). The C-positive heterochromatic regions assembled in some interstitial and subtelomeric chromosome regions, mostly in the centromeric/pericentromeric regions of all chromosomes, thus further highlighting the polymorphism in the 18th pair (Figure 2).

### 2.3. Chromosomal Location of 18S and 5S rDNA

The dual-color FISH experiments, using 5S rDNA and 18S rDNA probes, evidenced a divergent pattern among the *S. formosus* phenotypes. The 18S rDNA sequence was located exclusively in the 18th chromosomal pair in all five phenotypes (Figure 2 and Figure S2). With respect to the 5S rDNA, sites were located in the long arms (q arms) of two acrocentric pairs (15th and 19th) in the AG and GC phenotypes (Figure 2). In turn, the SR and HG phenotypes contained the same 5S rDNA sites described above, but also a small 5S rDNA site in the 18th acrocentric pair, which harbors the 18S rDNA cluster (Figure 2). The YS phenotype was the only one lacking 5S signals in the 19steoglossiformes chromosome pair, thus bearing solely 5S rDNA sites located in the q arms of the 15th chromosome pair. In addition, a remarkable polymorphism in size was found among the 18S rDNA clusters of the phenotypes, coinciding with the different sizes of their C-positive heterochromatin blocks. In this respect, the YS phenotype presented the largest block in their two equally large acrocentric chromosomes. The remaining phenotypes carry one large block and one small block in their unequal chromosomal pair, composed of one large and one relatively small acrocentric chromosome.

### 2.4. Chromosomal Location of SatDNAs of S. formosus

**Figure 3:**
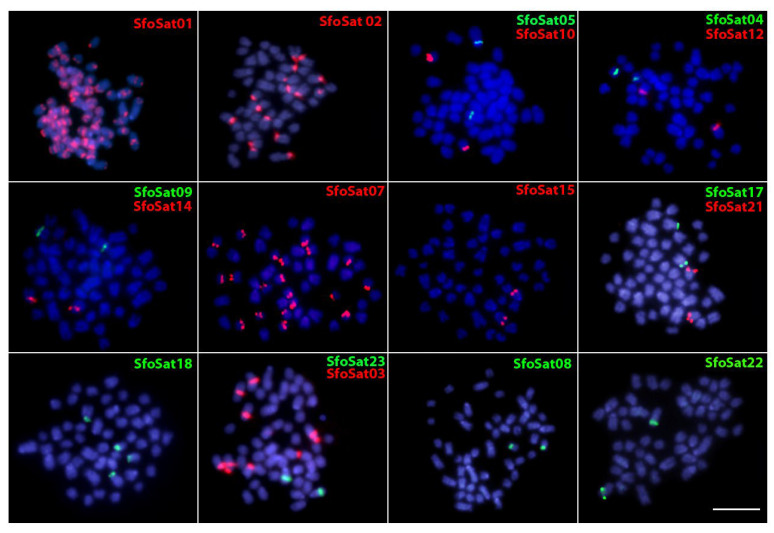
Image highlighting the chromosomal location of 17 SatDNAs using the metaphase plates of *S. formosus* (HG phenotype). The SatDNA family names are indicated on the left top, in green (ATTO488 labeled) or red (ATTO550 labeled). Bar = 5 μm.

In order to examine the chromosomal location of SfoSat DNAs we used both female and male mitotic metaphase plates of *S. formosus* (HG phenotype) in our two-color FISH experiments (Figure 3). Within the 17 successfully amplified SatDNAs families, we found positive hybridization signals in the centromeric and pericentromeric regions (SfoSat 01–05; SfoSat 07–10; SfoSat 12, 14, 17, 18) and, in some cases, in the interstitial (SfoSat 01) and telomeric/terminal regions (SfoSat 15, 21, 22, 23) (Figure 3). The SfoSat 01 hybridized in all chromosomes of *S. formosus*, while SfoSat 02, 03, 07, and 18 presented, respectively, signals in eight (16 st/a chromosomes), four (4 m/sm + 4 st/a chromosomes), 10 (20 st/a chromosomes), and two (4 st/a chromosomes) pairs (Figure 3). All the other 13 SatDNAs showed sites in only one chromosome pair. SfoSat 04, 08, and 10 showed signals in small st/a chromosomes, with SfoSat 09, 12, 14, 15, 17, and 21 in medium-sized st/a chromosomes, and SfoSat 05, 22, and 23 in large st/a chromosomes (Figure 3).

### 2.5. Whole Chromosome Paint Hybridization (WCP) and Comparative Genomic Hybridization (CGH)

**Figure 4:**
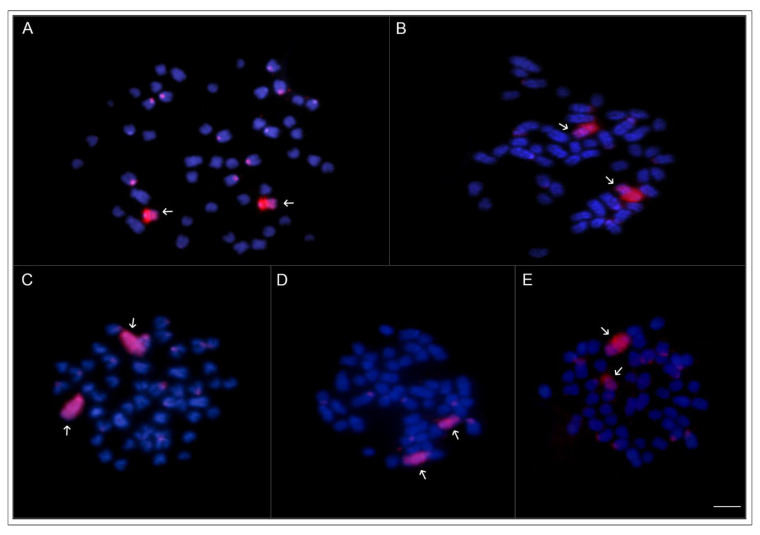
WCP experiments with SFO-A (arrows) painting probe applied on the metaphase plates of *S. formosus* SR (**A**); GC (**B**); HG (**C**); AG (**D**); and YS (**E**). Bar = 5 µm.

**Figure 5:**
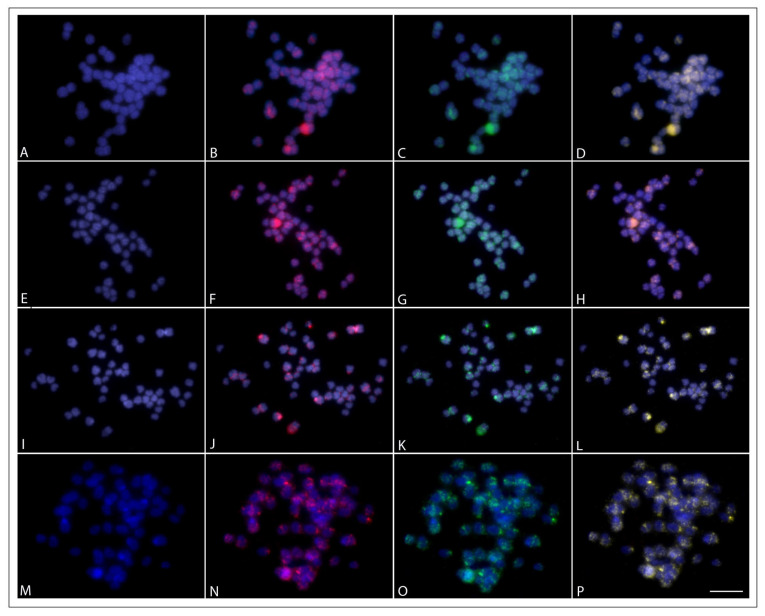
Comparative Genomic Hybridization, using metaphase plates of the SR phenotype (**A**,**E**,**I**,**M**), showing the co-hybridization with the gDNA of this same phenotype (**B**,**F**,**J**,**N**), labeled with Atto 550-dUTP, and the gDNAs of other phenotypes (**C**,**G**,**K**,**O**), namely, AG (**C**), GC (**G**), HB (**K**), and YS (**O**), labeled with Atto 488-dUTP. The fourth column (**D**,**H**,**L**,**P**) represents the resulting merged images. Bar = 5 µm.

Regarding the whole chromosome painting (WCP), the microdissected probe SFO-A, obtained from the 18th acrocentric pair of the YS phenotype, fully painted the 18th acrocentric pairs of the five phenotypes, revealing perfect similarity among them with a few unspecific centromeric/pericentromeric sites (Figure 4). The CGH experiments did not show color variant-specific regions between the SR and the other phenotypes (e.g., the 18th chromosomal pair NOR region). There were preferentially localized signals in most chromosomes’ centromeric/pericentromeric regions and some interstitial regions (Figure 5). Furthermore, each genomic hybridization method yielded results that were comparatively similar to each other, indicating that the color phenotypes had a low level of genome divergence.

## 3. Discussion

The 2n of *S. formosus* fits the range already observed for other Osteoglossidae species [21,35]. However, while *Osteoglossum* species possess higher chromosomal numbers (2n = 54–56) and karyotypes composed almost entirely of st/a chromosomes, the *Scleropages* species have a reduced number (2n = 44–50) and a higher number of bi-armed chromosomes, as observed in both Australian arowanas: *S. leichardti* with 2n = 44 (24 m/sm + 20 st/a) and *S. jardinii* with 2n = 48 (20 m/sm + 28 st/a), as reviewed in Cioffi et al. [21]. Furthermore, our results do not support the findings of Bian et al. [36] and Shen et al. [37] regarding the putative 2n = 48 chromosomes for *S. formosus*. Instead, our investigation demonstrates 2n = 50, as previously found by Urushido. [38] and Cioffi et al. [21].

The maintenance of a conserved macrokaryotype structure, as in *S. formosus*, is not an unusual occurrence, since different species can display a common 2n, as well as the same chromosomal features over a long evolutionary time. Indeed, the process of karyotype stasis (i.e., strong preservation of 2n and karyotype structure) has been already extensively reported [39,40,41,42]. This process is usually associated with frequent gene flow together with the absence of evolutionary barriers (e.g., ecological and geographic ones), but also other types of stabilizing selection mechanisms, thus allowing the preservation of well-established adaptations [41,43,44,45]. For instance, the Eupercaria, a very rich marine species group is characterized by sharing an extensive 2n = 48 and a karyotype composed entirely of acrocentric chromosomes [45]. In turn, this is not an exclusive characteristic for animals, but also for plants species, as observed in, e.g., *Pachycladon*, a famous genus for its island radiation, in which all the extant species present 2n = 20 chromosomes [40]. In this sense, the chromosomal number we observed for the five *S. formosus* phenotypes could be explained, in general, by (i) a relatively recent divergence time of populations, (ii) a geographic distribution allowing the sharing of gene pools (i.e., as seen in sympatric populations), thus enabling a stable gene flow, and/or (iii) a cohort of evolutionary forces (e.g., genetic drift, natural selection) preventing the survival of conspicuous karyotype changes and events of directional selection (see [43]). As described by Yue et al. [10], most color varieties are isolated in different geographic locations (except for the green phenotypes), thus representing allopatric populations (see [14] and references therein). Therefore, it is most likely that a recent divergence time prevented the accumulation and fixation of significant 2n changes, as found in *S. formosus*.

Ribosomal DNA mapping has been extensively used in many recent cytogenetic investigations (reviewed in [46]), constituting a powerful molecular marker for taxonomic issues [47,48,49,50], intra-/interspecific chromosomal rearrangements [49,51,52], and sex chromosome dynamics and differentiation processes [53,54]. In this study, we were able to highlight three distinct patterns of the 5S rDNA distribution: AG and GC phenotypes with four sites, SR and HG phenotypes with six sites, and the YS with only two sites. Also identified was a major chromosomal polymorphism (see results, in the chromosomal mapping of ribosomal DNA section) involving the accumulation of the 18S rDNA and constitutive heterochromatin, which appears to be associated with specific phenotypes.

**Figure 6:**
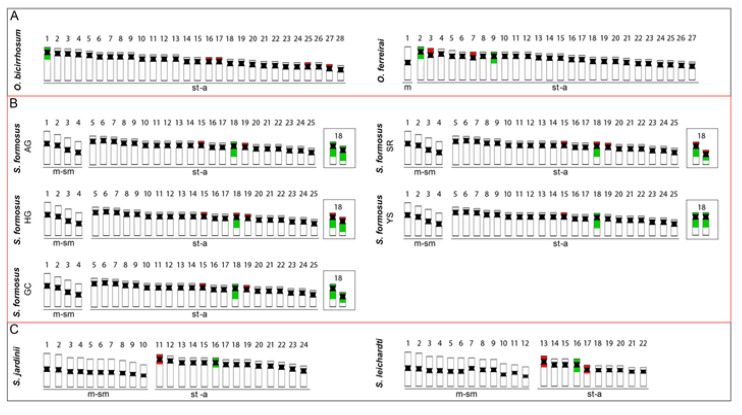
Representative ideograms of arowana species. (**A**) The south american *Osteoglossum*; (**B**) The asiatic *S. formosus* and (**C**) The Australian *Scleropages*) showing the distribution of the 18S (green) and 5S (red) rDNA sites on chromosomes, based on the present data and some other previous ones [21]. The *S. formosus* color variants analyzed in this study are highlighted in red.

Overall, the distribution of the 18S rDNA seems to be relatively uniform among *Osteoglossum* and *Scleropages* chromosomes, with only two sites, with a single exception so far found in *O. ferreirai*, which bears four 18S rDNA sites (Figure 6). On the other hand, the 5S rDNA clusters appear to have a major dynamic behavior in Osteoglossinae, ranging from two to eight sites and participating in a linkage group with the 18S rDNA in the polymorphic 18steoglossiformes pair of *S. formosus* (Figure 6). Repetitive DNA (e.g., DNA satellites and multigene families) are putatively regulated by a concerted evolution [55], in which DNA sequences of a specific genomic region (e.g., repetitive in tandem sequences) act as a unit to promote or prevent mutations. Thus, a mutation in one repetitive unit may promote a series of the same mutation in the other units. This type of mechanism relies on well-established molecular processes, such as DNA repair machinery (i.e., through homologous and non-homologous recombination), gene conversion, and transposon activity [55,56], all capable of carrying ribosomal DNA sites into other autosomal chromosomes. In the case of the 5S rDNA, although homologous recombination may act as a possible carrying mechanism, it is possible that the centromeric/pericentromeric sites of *Scleropages* could have facilitated transposition events. Indeed, such sequences (e.g., transposons and retrotransposons) have already been found in centromeric clusters associated with other ribosomal genes (see [52,57,58]).

Bian et al. [36] proposed that *S. formosus* may have a ZW sex chromosome system. However, even employing several molecular cytogenetic techniques, including WCP and CGH, we found no indication of sex chromosomes in this species, which supports the earlier findings by Cioffi et al. [21]. As a matter of fact, its putative ZW system is more likely the polymorphic pattern that occurs in the 18th chromosome pair, including the accumulation of constitutive heterochromatin and 18S rDNA (Figure 2). Similar chromosome polymorphisms have been reported for other fish species [59,60,61], and they are generally thought to result from copy number variations caused by uneven crossing over, transposon activity, or dosage compensation mechanisms [46,62,63]. Therefore, while conserved in their macrostructure, the karyotypes of the *S. formosus* color phenotypes have considerable variability in some multigene DNA families, which could eventually lead to well-established evolutionary lineages.

Our satellitome analysis in *S. formosus* represents the first one for an Osteoglossiformes species. We found 25 SatDNA families in the genome of *S. formosus*, of which 17 were successfully amplified and hybridized in the chromosomes of male and female individuals. When compared to other ray-finned fishes, these results indicate a reduced number of SatDNAs families in *S. formosus*. Indeed, recent investigations [33,34,64] have characterized cases of greater SatDNAs diversity, as found in characins *Triportheus auritus* and *Astyanax paranae*, which bear, respectively, 53 and 64 SatDNAs families, and the singular case of *Megaleporinus macrocephalus*, which possesses more than 100 different SatDNAs families. Currently, there are two non-excluding putative scenarios explaining the formation and evolution of SatDNAs: i) the independent origin of new families and ii) the phylogenetic sharing of SatDNAs in closely related lineages [26,65,66]. In the first case, “de novo” nucleotide duplications and/or transposition events arise in euchromatic regions of the genome, giving birth to new SatDNA families which can be spread among different chromosomes. In the second case, it is understood that the genome of each lineage presents a library of ancestral SatDNA sequences (the “library hypotheses”), and these sequences can expand or contract in new generations [55]. As observed in some other ancient lineages, such as sturgeons, the shortened copy number and diversity of repetitive DNAs are explained by a decreased rate of molecular evolution [67], thus preventing the fixation of mutations and transposition events, which are important stepping stones for the formation of new SatDNAs [68,69]. The rate of molecular evolution in *S. formosus* is comparatively lower than those found in other Teleostei [70], which could explain the differences found in the number of SatDNAs retrieved by our investigation and the lack of shared sequences revealed by BLAST search. Alternatively, it is also possible that the genome size difference between *S. formosus* and other Teleostei is responsible for the reduced SfoSatDNAs diversity, in which smaller genomes possess, consequently, fewer SatDNAs families.

Repetitive elements account for the majority of DNA elements in eukaryotic genomes [71]. The study of these sequence elements is required to understand the nature and significance of genome size variation between species as well as the comprehensive structure and evolution of fish genomes. The fastest-evolving DNA sequences in genomes, centromeric repeats have been shown to differ between populations or even between closely related species, as was the case in *Drosophila* [72] and Macropodine marsupials [73]. Our findings suggest that the SfoSat 01, 02, 03, and 07, which were found to be located in the pericentromeric regions of nearly all chromosomes, may be significant for *S. formosus*’ centromeric activity. Some SatDNAs are predicted by [74] to contribute to centromeric function. Similar outcomes in *Triportheus* species have recently been discovered [33]. Once it was demonstrated that divergence in centromeric sequences may lead to reproductive isolation and, eventually, species radiation, several authors hypothesized the significance of this fast mechanism in the speciation process [72,75].

It is worth noting that there has been an increasing amount of research in the literature showing how SatDNAs play a role in centromere epigenetics as well as chromosome speciation [26,73,75,76]. These authors described numerous instances in which important centromere features, such as CENP-B and/or CENP-A DNA motifs, are incorporated into the structure of SatDNAs or even examples in which SatDNA transcripts, such as miRNA and siRNA, regulate pericentromeric heterochromatin and gene expression. The creation of kinetochores and the posterior attachment of the spindle fibers are both processes that the centromere participates in, making it an essential component of the genome and required for the proper disjunction of chromosomes [72,76]. As *S. formosus* SatDNAs have a similar distribution, this suggests that their functional significance in the development and control of centromeres. The detection of homologous chromosomes during meiosis can be affected by changes in SatDNA structure, and over time, these changes can cause post-zygotic barriers to emerge, which results in differing patterns of SatDNAs in certain lineages [76].

We found evidence for population genetic structure and microstructure variations in the karyotypes of *S. formosus* color phenotypes. However, the results do not fully support the existence of distinct lineages, or evolutionary units, that correspond to different color phenotypes in *S. formosus*. Therefore, future studies should include a dense sampling of natural populations and combine genomic approaches with cytogenetic, morphological, and ecological data to better delimit the taxonomic boundaries of these variants using formal species delimitation approaches. Such advances will be important to successfully understand the ecology, life history, and diversity of *S. formosus*, allowing the development of appropriate conservation actions for this endangered species.

## 4. Materials and Methods

### 4.1. Individuals and Conventional Cytogenetics

The sampling individuals are presented in Table 2. Because of their status as a critically endangered taxon, commercial trading of *S. formosus* is allowed only for captive-bred, F2-generation individuals. Accordingly, 22 individuals from an aquarium trade in Thailand were legally collected (Table 2), and a certificate of parental origin accompanied each one of them. This sample represents five different phenotypes of the three naturally occurring varieties of *S. formosus*: Super Red (SR—red variety), Gold Crossback (GC—golden variety), Highback Golden (HG—golden variety), Asian Green (AG—green variety) and Yellow-Tail Silver (YS—green variety). We used the caudal fin regeneration method for chromosome preparation [77], with adjusted regeneration timeframes (ranging from 5 to 10 days), to obtain mitotic chromosomes without the need to sacrifice the specimens. The chromosomes were stained with a 10% Giemsa solution (pH 6.8), and the constitutive heterochromatin was detected according to the C-banding procedure [78].

### 4.2. DNA Extraction and Genome Sequencing

We extracted the genomic DNAs (gDNAs) from the fins tissues of one individual of each phenotype, and one specimen of the Highback Golden phenotype was selected for the genome sequencing. The extraction procedure followed the standard phenol-chloroform-isoamyl alcohol method [79]. The low-pass shotgun sequencing (2 × 150 bp paired-end) was performed on the BGISEQ-500 platform at BGI (BGI Shenzhen Corporation, Shenzhen, China), yielding 2.14 Gb. Raw reads are available in the Sequence Read Archive (SRA-NCBI) under the accession number SRR23609111.

### 4.3. Bioinformatic Analyses

Initially, we performed a quality filtering of reads using Trimmomatic software [80]. Then, the satellitomes were characterized using a combination of custom python scripts (https://github.com/fjruizruano/satminer, accessed on 15 January 2023) and the TAREAN tool [27]. Specifically, we start with a characterization of SatDNAs in the TAREAN tool in a random selection of 2 × 500,000 reads. Then, we filtered out the identified SatDNAs with DeconSeq [81] and repeated these steps until no SatDNA was identified. Next, we removed other tandemly repeated sequences commonly outputted by TAREAN, such as multigene families, from the catalog. Finally, we performed a homology search using the RepeatMasker 4.1.5 software (https://github.com/fjruizruano/satminer/blob/master/rm_homology.py, accessed on 23 February 2023) to group sequences into variants (>95% of similarity), family (between 80 and 95% of similarity), and superfamily (between 50 and 80% of similarity), as proposed by [28].

After that, the abundance and divergence values of SatDNAs were estimated using RepeatMasker software [82]. For this, we selected 2 × 7,125,600 reads and aligned them against the SatDNA catalog with a custom python script (https://github.com/fjruizruano/ngs-protocols/blob/master/repeat_masker_run_big.py, accessed on 23 February 2023). The abundance of each satellite DNA was estimated as the quotient of the number of mapped reads and the number of analyzed nucleotides. Then, we named SatDNA families in decreasing order of abundance, as suggested by Ruiz-Ruano et al. [28]. Additionally, we BLAST-searched [83] the satellitome of *S. formosus* against the GenBank/NCBI nucleotide database to verify the occurrence of conserved SatDNAs.

### 4.4. Primer Design and DNA Amplification via Polymerase Chain Reaction (PCR)

We designed primers for 24 out of the 25 sSatDNAs that were characterized (marked with an asterisk in Table 1). The PCR procedures used the optimal amplification temperatures and DNA template concentrations for each SatDNA, according to [33]. The following cycles were used for each sequence: initial denaturation at 95 °C for 5 min, 30 cycles with denaturation at 95 °C for 20 s, annealing at 52 °C to 60 °C for 40 s, extension at 72 °C for 30 s, and final extension at 72 °C for 10 min. The PCR products were checked by electrophoresis on 2% and 1% agarose gels to validate the amplification and check the integrity of the SatDNAs. Finally, they were quantified using the NanoDrop spectrofotometer (ThermoFisher Scientific, Branchburg, NJ, USA).

### 4.5. Fluorescence in Situ Hybridization (FISH)

We performed fluorescence in situ hybridization (FISH) using probes derived from the SatDNA’s PCRs and from the 5S and 18S ribosomal DNAs (rDNA) to detect potential polymorphisms related to the chromosomal location of ribosomal DNA genes and to characterize the location of the satellitome of *S. formosus*. The probes of the 5S and 18S rDNA were previously amplified via PCR from the nuclear genome of *Hoplias malabaricus* [84,85] and cloned into plasmid vectors and propagated in competent cells of *Escherichia coli* DH5α (Invitrogen, San Diego, CA, USA). The 5S probe corresponded corresponds to the 5S rRNA coding region, comprising 120 base pairs (bp) associated with a non-transcribed spacer, NTS [86]. The 18S probe corresponds to a 1400 bp segment of this rRNA gene. The 5S rDNA probe was labeled with Atto-550-dUTP (Red fluorescence) and the 18S rDNA probe with Atto-488-dUTP (Green fluorescence). From the total of 25 SatDNAs, we were able to successfully amplify 17 sequences, which were labeled for FISH experiments with Atto-550-dUTP or Atto-488-dUTP. All probes used were labeled using a nick-translation labeling kit from Jena Bioscience (Jena, Germany), in accordance with the manufacturer’s manual. The FISH procedure was conducted under high-stringency conditions, as described in Yano et al. [87], and all metaphase plates were stained with 4′,6-diamidino-2-phenylindole (DAPI) solution.

### 4.6. Microdissection and Preparation of Chromosome Painting Probes

Twelve copies of the 18th acrocentric pair of the Yellowtail Silver (YS) phenotype of the green variety, which harbors two equally large acrocentric chromosomes, were manually microdissected using a glass needle, in order to look for chromosomal homologies among the color variants connected to a putative ZW-pair [36]. The material was then amplified using a degenerate oligonucleotide-primed polymerase chain reaction (DOP-PCR) procedure, described in Yang et al. [88]. We named the microdissected probe SFO-A (SFO: *Scleropages formosus*; A: largest acrocentric pair) and properly labeled it with Spectrum-Orange-dUTP (Vysis, Downers Grove, IL, USA) in a secondary DOP-PCR, using 1 μL of the primarily amplified product as DNA template [88]. Chromosome preparations of all five phenotypes were then used for whole chromosome painting (WCP) procedures, following the protocol of Yano et al. [87].

### 4.7. Comparative Genomic Hybridization (CGH)

To check the degree of genomic divergence present among the color variants, we co-hybridized the gDNA of each specimen with the gDNA of the Super Red (SR) phenotype of the red variety, which was also the phenotype we used as background chromosomes for visualization of hybridization patterns. We labeled the SR gDNA directly with Atto-550-dUTP, while the gDNA of the other phenotypes were labeled with Atto-488-dUTP. In all experiments, we blocked common genomic repetitive sequences using C0t-1 DNA (i.e., a fraction of genomic DNA enriched for highly and moderately repetitive sequences), prepared from each *S. formosus* phenotype following the protocol of Zwick et al. [89]. The final hybridization mixture (20 μL for each slide) was composed of 500 ng of SR gDNA, 500 ng of the compared gDNA phenotype, and 15 μL of unlabeled C0t-1 DNA of the compared phenotype mixed together in a hybridization buffer containing 50% of formamide, 2x SSC, 10% SDS, 10% dextran sulfate, and Denhardt´s reagent (pH = 7.0). The ratio of the probes versus the C0t-1 DNA was based on previous experiments we have performed in fishes [20,21,24], and the CGH procedure followed that outlined in Symonová et al. [90].

### 4.8. Microscopy and Image Processing

We analyzed >30 metaphase spreads per individual to assess the diploid number (2n), karyotype structure, and FISH results. The images were captured using an Olympus BX50 microscope (Olympus Corporation, Ishikawa, Japan) with CoolSNAP, and the images were processed using the Image-Pro Plus 4.1 software (Media Cybernetics, Silver Spring, MD, USA). We classified chromosomes as metacentric (m), submetacentric (sm), subtelocentric (st), or acrocentric (a) according to their arm ratios [91]. Finally, we assembled schematic representations to demonstrate the chromosomal distribution of the 5S and 18S rDNA sequences in different species of Osteoglossidae, using data from this study and from [21].

## Figures and Tables

**Table 1:** Main characteristics of 25 SatDNAs found in *S. formosus*. Abundance is given as the proportion of the satellite DNA in the analyzed libraries. * Indicates the SatDNAs chosen for FISH experiments. SF = superfamily; RUL = repeat unit lengths.

SatDNA Family	SF	RUL	Abundance	Divergence	A + T (%)
* SfoSat01-180		180	0.025654546	7.06	36.7
* SfoSat02-39		39	0.016109651	11.84	56.4
* SfoSat03-198		198	0.010209052	2.27	52.5
* SfoSat04-109		109	0.001647104	1.88	59.6
* SfoSat05-107		107	0.001390976	7.70	55.1
SfoSat06-4000		4000	0.001339709	7.11	51.4
* SfoSat07-156		156	0.001159315	2.80	57.1
* SfoSat08-111		111	0.001183768	6.09	60.4
* SfoSat09-108		108	0.000889043	11.46	64.8
* SfoSat10-239		239	0.00089077	3.73	58.6
SfoSat11-1682		1682	0.000778082	9.24	50.2
* SfoSat12-337		337	0.000767312	6.28	58.8
SfoSat13-2034		2034	0.000692241	9.07	56.8
* SfoSat14-95		95	0.000607639	1.61	61.1
* SfoSat15-261		261	0.000255594	3.65	50.6
SfoSat16-6		6	0.000252204	15.04	50.0
* SfoSat17-699		699	0.000223762	2.97	50.6
* SfoSat18-767		767	0.000166812	4.07	43.0
SfoSat19-993		993	0.00012931	1.61	40.7
SfoSat20-677		677	0.000127314	0.87	54.2
* SfoSat21-651	1	651	0.000115523	4.55	38.2
* SfoSat22-293		293	0.000112537	3.07	53.9
* SfoSat23-291	1	291	0.000103291	5.61	50.5
SfoSat24-120		120	0.000099403	5.08	60.0
SfoSat25-616		616	0.000089154	1.77	55.9

**Table 2:** Species, sampling origin, and number of individuals analyzed.

Species (Variety)	Phenotype (Code)	Sampling Site	n
*S. formosus* (green)	Asian Green (AG)	Aquarium trade, Song Khram river	(02♀02♂)
*S. formosus* (green)	Yellow TailSilver (YS)	Aquarium trade, Song Khram river	(04♀02♂)
*S. formosus* (golden)	Gold Crossback (GC)	Aquarium trade, Origin unknown	(02♀02♂)
*S. formosus* (golden)	High Back Golden (HG)	Aquarium trade, Origin unknown	(02♀02♂)
*S. formosus* (red)	Super Red (SR)	Aquarium trade, Origin unknown	(03♀02♂)

## Data Availability

The datasets generated during and/or analyzed during the current study are available from the corresponding author on reasonable request. The datasets generated and analysed during the current study are available in the GenBank repository, under accession numbers OQ743811-OQ743835.

## Supplementary Materials

The following supporting information can be downloaded at: https://www.mdpi.com/article/10.3390/ijms24109005/s1.
