# Supplementary figures and images for: Cytogenetics Meets Genomics: Cytotaxonomy and Genomic Relationships among Color Variants of the Asian Arowana Scleropages formosus
Source: Int J Mol Sci. 2023 May 19;24(10):9005. doi: 10.3390/ijms24109005 (PMC10219274; doi:10.3390/ijms24109005)

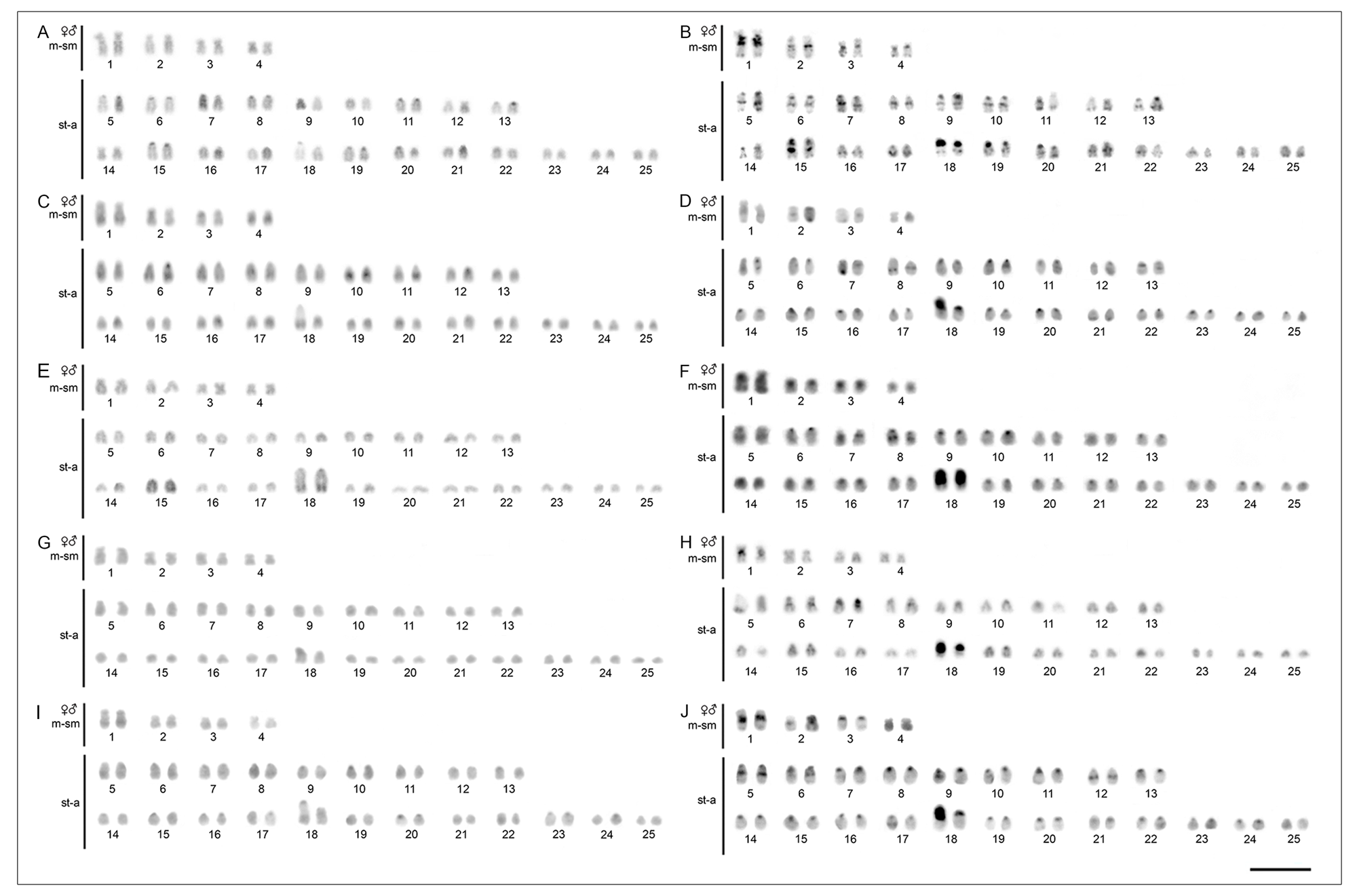

Supplement: Supplementary file 1 [file ijms-24-09005-s001.zip › Sup Fig S1.tif]

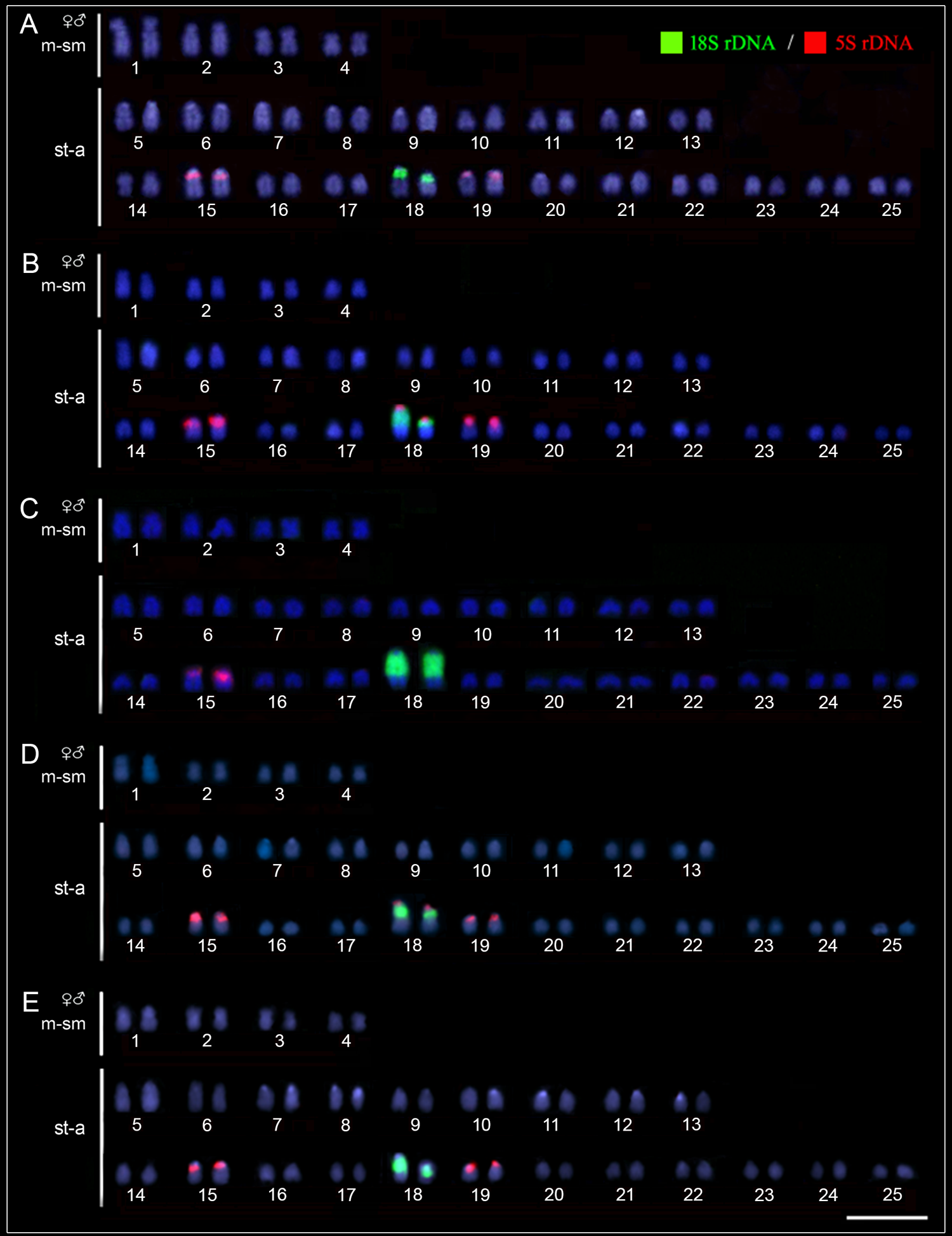

Supplement: Supplementary file 1 [file ijms-24-09005-s001.zip › Sup Fig S2.tif]
